# Chondrocyte‐to‐osteoblast transformation in mandibular fracture repair

**DOI:** 10.1002/jor.24904

**Published:** 2020-11-18

**Authors:** Sarah A. Wong, Diane P. Hu, Joshua Slocum, Charles Lam, Michael Nguyen, Theodore Miclau, Ralph S. Marcucio, Chelsea S. Bahney

**Affiliations:** ^1^ Department of Orthopaedic Surgery, Orthopaedic Trauma Institute University of California 2550 23rd Street Building 9, 3rd Floor San Francisco California 94110 USA; ^2^ Oral and Craniofacial Sciences Program, School of Dentistry University of California San Francisco California USA; ^3^ Steadman Philippon Research Institute Center for Regenerative Sports Medicine 181 W Meadows Drive Vail Colorado 81657 USA

**Keywords:** cartilage grafts, endochondral ossification, fracture repair, hypertrophic chondrocytes, mandible fracture, transdifferentiation, transformation

## Abstract

The majority of fracture research has been conducted using long bone fracture models, with significantly less research into the mechanisms driving craniofacial repair. However, craniofacial bones differ from long bones in both their developmental mechanism and embryonic origin. Thus, it is possible that their healing mechanisms could differ. In this study we utilize stabilized and unstabilized mandible fracture models to investigate the pathways regulating repair. Whereas fully stable trephine defects in the ramus form bone directly, mechanical motion within a transverse fracture across the same anatomical location promoted robust cartilage formation before boney remodeling. Literature investigating long bone fractures show chondrocytes are a direct precursor of osteoblasts during endochondral repair. Lineage tracing with Aggrecan‐Cre^ERT2^::Ai9 tdTomato mice demonstrated that mandibular callus chondrocytes also directly contribute to the formation of new bone. Furthermore, immunohistochemistry revealed that chondrocytes located at the chondro‐osseous junction expressed Sox2, suggesting that plasticity of these chondrocytes may facilitate this chondrocyte‐to‐osteoblast transformation. Based on the direct role chondrocytes play in bone repair, we tested the efficacy of cartilage grafts in healing critical‐sized mandibular defects. Whereas empty defects remained unbridged and filled with fibrous tissue, cartilage engraftment produced bony‐bridging and robust marrow cavity formation, indicating healthy vascularization of the newly formed bone. Engrafted cartilage directly contributed to new bone formation since a significant portion of the newly formed bone was graft/donor‐derived. Taken together these data demonstrate the important role of chondrocyte‐to‐osteoblast transformation during mandibular endochondral repair and the therapeutic promise of using cartilage as a tissue graft to heal craniofacial defects.

## 1 INTRODUCTION

Craniofacial bones differ from appendicular bones in both their embryonic origin and developmental mechanism.^1^ Appendicular bones are derived from lateral plate mesoderm through the process of endochondral ossification. This process begins with the differentiation of mesenchymal stem cells into chondrocytes that form a cartilage template in the approximate size and shape of the future bone. As the skeleton develops, the cartilage anlage is eventually converted to bone.^2^ In contrast, craniofacial bones develop from the cranial neural crest through the process of intramembranous ossification. Unlike endochondral ossification, this process does not involve a cartilage intermediate. Rather, osteochondral progenitors directly differentiate into osteoblasts to form bone.^1^ The majority of mechanistic fracture research has been focused on long bone fractures in the appendicular skeleton. Given the significant developmental difference between craniofacial and long bones, it is possible that these bones differ in their healing mechanisms as well. To optimize current treatments and develop novel therapies for craniofacial fractures, improving our mechanistic understanding of specific fracture types is critical.

Long bone fractures heal through the combination of intramembranous and endochondral ossification, with local mechanical stability generally believed to be the fundamental factor in driving the mechanism of healing.^3^, ^4^, ^5^ A relatively high amount of mechanical motion between the fractured bone ends promotes cartilage formation and endochondral repair, whereas rigid stability promotes direct bone formation through intramembranous ossification.^6^ Recent genetic evidence from long bone fracture studies demonstrate that chondrocytes within the provisional fracture callus matrix directly contribute to the formation of bone by transforming into osteoblasts.^7^, ^8^, ^9^, ^10^, ^11^, ^12^, ^13^, ^14^ There is limited research into the molecular mechanisms by which chondrocytes transform into osteoblasts, but recent data suggest hypertrophic chondrocytes gain cellular plasticity in response to the invading vasculature through the expression of programs associated with stemness (Sox2, Oct4, Nanog), and then undergo osteogenesis through activation of the Wnt/β‐catenin signaling pathway.^7^, ^8^, ^11^, ^13^, ^14^ Importantly, these new studies update our mechanistic understanding of long‐bone fracture repair and can be used to develop novel approaches to stimulate fracture repair by promoting the process of endochondral ossification.^15^

While much less frequently studied, the mandible is one of the most commonly fractured craniofacial bones, sustaining up to 70% of all maxillofacial fractures.^16^ Its daily functional requirement and esthetic role necessitate the development of new and improved therapies. Although the mandible develops via intramembranous ossification, it has been shown to heal similarly to long bone fractures with a combination of intramembranous and endochondral ossification.^17^ In this study we aimed to study the role of chondrocyte‐to‐osteoblast transformation during mandible fracture repair by utilizing two mandible fracture models: a stabilized trephine defect model that heals via intramembranous ossification, and an unstabilized osteotomy that heals via endochondral ossification.

In addition to building new knowledge about the molecular and cellular mechanisms of mandible fracture repair, this study also aimed to explore endochondral routes of promoting mandibular bone regeneration. Autologous bone grafts are the current standard of care for improving healing in clinical cases of malunion or in large bone defects in both long bone and mandibular fractures. While clinical outcomes of autologous bone grafts are generally favorable, they are associated with significantly increased risk to the patient. Autograft procedures typically require a second surgical site, increased surgical time, increased blood loss and are associated with high rates of donor site morbidity.^11^, ^18^ Alternatively, bone allografts are readily available, but they have significantly reduced bioactivity and a high rate of clinical failure due to poor osteointegration and osteonecrosis of the graft.^19^ Thus, the discovery and development of alternative graft tissues and materials would be clinically beneficial. Unlike bone, cartilage is an avascular tissue that is not only capable of surviving without a vascular supply but is also responsible for recruiting blood vessel invasion for the newly formed bone during endochondral ossification.^2^, ^3^ Here we test the efficacy of cartilage grafts in healing mandibular defects using critical‐sized versions of the mandible fracture models described. The central hypothesis of this study is that, despite different developmental pathways, mandible fracture healing parallels long bone repair with cartilage directly contributing to the formation of new bone during native healing or with transplantation.

## 2 METHODS

### 2.1 Fractures

**Figure 1:**
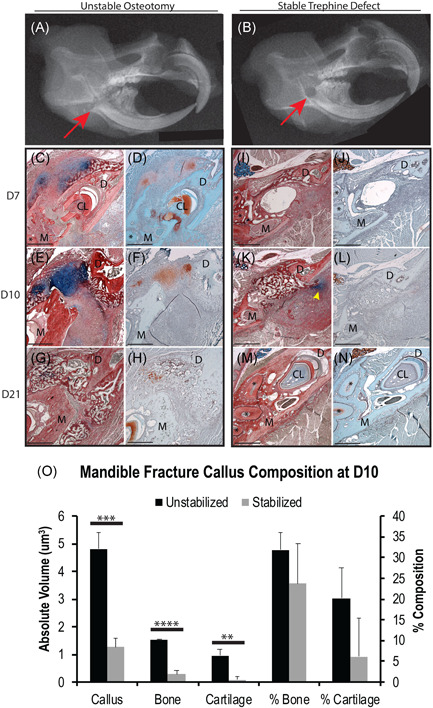
Fracture stability mediates mandibular ossification mechanisms. X‐ray images of an unstable transverse osteotomy (A) and stable 1 mm trephine defect (B) confirm the proper anatomical location of fracture site (red arrow) within the right mandibular ramus of C57BL/6 mice. Mandibles were imaged at 7 days postfracture (*N* = 5/group). HBQ (C, E, G, I, K, M) and Saf‐O (D, F, H, J, L, N) standard histology staining of transverse sections reveals that mandibles with unstable osteotomies (C–H) develop a robust cartilage callus and primarily heal through endochondral ossification, whereas mandibles with stable trephine defects (I–N) develop little to no cartilage and primarily heal through intramembranous ossification. Small amounts of cartilage were found adjacent to the fracture site in the stabilized fracture model at 10 days postfracture, usually ventral and posterior to the defect (K, yellow arrowhead). Cartilage was only observed in the stable fracture model at day 10 postfracture and was most‐likely due to motion derived from residual bone chips left behind from drilling the trephine defect. Stereological analysis of callus tissue composition confirmed that the absolute volume of cartilage was significantly less in stable than in unstable fractures at D10, which is the time point during which the greatest amount of cartilage was observed in either model (O). Complete bony‐bridging was observed in the unstable model at D21 postfracture (G, H) and in the stable model at D10 postfracture (K, L). HBQ: cartilage is blue, bone is red. Saf‐O: cartilage is red, bone is teal. (*) in histology = mandibular molar roots. *N* = 5/time point/fracture type. Scale = 500 µm. **p < .01. ***p < .001. ****p < .0001. CL, cervical loop; D, distal; HBQ, hall brunt quadruple; M, mesial; Saf‐O, Safranin‐O [Color figure can be viewed at wileyonlinelibrary.com]

**Figure 2:**
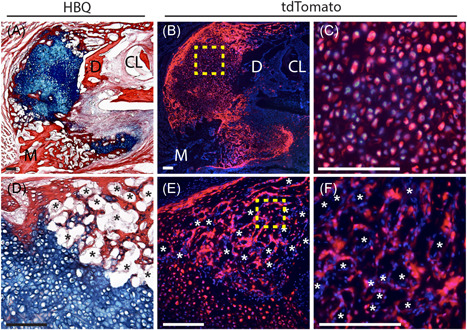
Fracture callus chondrocytes give rise to osteoblasts and bone‐lining cells during mandibular endochondral repair. Lineage tracing of mandibular fracture callus chondrocytes was performed using the Aggrecan‐Cre^ERT2^driver to induce cartilage‐specific Cre recombination in the stop‐floxed Ai9 tdTomato reporter mouse. Cre recombination was induced with daily Tamoxifen injections from days 6‐10 postfracture with samples harvested at 14 days postfracture. Fluorescence microscopy indicates robust tdTomato expression in chondrocytes within the fracture callus indicating highly efficient Cre recombination (C, boxed region in B). Robust tdTomato expression is also observed in osteoblasts/cytes within the newly formed bone and in bone‐lining cells, confirming their chondrocyte‐derivation (F, boxed region in E). Low‐magnification images demonstrate the significant contribution of chondrocytes to new‐bone formation (A, D correspond to B, E). DAPI counterstain was used to visualize nuclei (blue). *Blood vessels. *N* = 5. Scale = 100 µm. CL, cervical loop; D, distal; DAPI, 4′,6‐diamidino‐2‐phenylindole; M, mesial [Color figure can be viewed at wileyonlinelibrary.com]

All studies were approved by the UCSF Institutional Animal Care and Use Committee and the results have been reported according to ARRIVE guidelines. The following mice were obtained from Jackson Labs and maintained in our colony: C57BL/6J (Stock #: 000664), Aggrecan‐Cre^ERT2^ (Stock #: 019148), Ai9 tdTomato HZE (Stock #: 007909), LacZ reporter (Stock #: 002073), Nu/J (Stock #: 002019), GFP reporter (Stock #: 003291). Mice were anesthetized with a 1:1 mixture of ketamine (60 mg/kg) and dexmedetomidine (0.3 mg/kg) delivered intraperitoneally.^8^, ^11^ Mandible fractures with different levels of stability were generated in adult (10–16 week) male mice by creating either (1) an unstable osteotomy through the right mandibular ramus from the anterior border of the coronoid process to the anterior border of the angular process (Figure 1A) or (2) by drilling a 1‐mm diameter hole to form a trephine defect through the right mandibular ramus (Figure 1B). In the unstable model, no bone was resected and fractured bone ends were allowed to oppose one another. No external or internal fixation was used to stabilize this model to reduce irritation of the surrounding muscle and to promote robust cartilage callus formation. Animals were randomly assigned to fracture group, and the fracture site location was confirmed via radiography. Animals were revived using an atipamezole reversal agent (6 mg/kg) and were given subcutaneous buprenorphine analgesic (0.05–0.1 mg/kg) immediately post‐op, and at 4 and 24 h postfracture. Animals received prophylactic antibiotic for up to 5 consecutive days following surgery (25 mg/kg cefazolin or 15 mg/kg enrofloxacin). Any animals displaying signs of infection were excluded from this study. Animals were fed a soft food diet for 7 days and monitored closely for pain and weight loss. Mice were socially housed (up to five animals per cage) and allowed to ambulate freely until experimental endpoints.

### 2.2 Histology

Mandibles were harvested 7, 10, or 21 days postoperatively and fixed for up to 24 h at 4°C in 4% paraformaldehyde (pH 7.4), decalcified for four weeks using 19% EDTA (pH 7.4), paraffin‐embedded and sectioned (10 μm). Standard histological staining protocols were used to visualize cartilage and bone at each time point: hall brunt quadruple (HBQ; bone stains red, cartilage stains blue); Safranin‐O/Fast Green (cartilage stains red, green counterstain) (*N* = 5/time point/fracture type).

### 2.3 Stereology

Quantification of callus size and composition (cartilage, bone, other) was determined using an Olympus CAST system and software by Visiopharm according to established methodologies.^20^ HBQ stained serial sections through the entire fracture site, spaced 300 µm apart were analyzed. The fracture callus was outlined using low magnification (×20, ×2 objective with ×10 ocular magnification) to determine the region of interest. Twenty percent of this region was quantified using automated uniform random sampling to meet or exceed the basic principles of stereology.^20^ Cell and tissue identity within each random sampling domain was determined at high magnification (×200, ×20 objective with ×10 ocular magnification) according to histological staining patterns and cell morphology. Absolute volumes of specific tissues (e.g., bone or cartilage) were determined using the Cavalieri formula and these absolute volumes were used to determine the relative percent composition of tissues within the fracture callus.^20^ Total fracture callus volume was measured as callus points.

### 2.4 Immunohistochemistry

Immunohistochemistry for Sox2 (1:250; ab107156; from the Abcam kit) and Osteocalcin (OC; 1:250; ab93876; Abcam) was performed on paraffin‐embedded serial sections of samples harvested 10 days postfracture (*N* > 5 per antibody). The basic protocol included antigen retrieval in 10 mM sodium citrate buffer (20 min, 100°C), endogenous peroxidase blocking in 3% H_2_O_2_ (30 min, room temperature) and nonspecific epitope blocking with 5% goat serum (GS; 1 h, room temperature). Primary antibodies were applied to sections overnight at 4 C. An horseradish peroxidase‐conjugated, species‐specific secondary antibody (AB307P; Millipore; 1:200 in phosphate‐buffered saline with 5% GS, 1 h, room temperature) was detected using 3,3’‐diaminobenzidine colorimetric reaction. Cell counting of randomly selected ×20 fields of view was performed to determine the % positively staining chondrocytes at the transition zone and at the center of the cartilage callus for both antibodies (*N* = 5/antibody). The callus was divided into the central callus versus the transition zone using the cellular morphology and regional demarcations described previously.^8^

### 2.5 Lineage tracing

Aggrecan‐Cre^ERT2^ and Ai9 tdTomato HZE mice were bred so that their progeny were heterozygous for Cre and tdTomato reporter alleles. Genotype was confirmed through gel electrophoresis according to Jackson Lab's genotyping protocols. Unstable mandible fractures were created as above. Cre recombination was induced with daily injections of Tamoxifen (75 mg/kg) from days 6 to 10 postfracture.^8^ This recombination period aligned with the peak of the soft, cartilaginous phase of mandibular fracture repair. Samples were harvested 14 days postfracture, fixed and decalcified as above, cryoembedded, and sectioned (10 μm). Samples were mounted in VectaShield with DAPI (H‐1200; Vector) and visualized using an epifluorescence microscope (*N* = 5).

### 2.6 Cartilage grafts

Critical‐sized defects were created in the right mandibular ramus of immunocompromised Nu/J mice either by creating two parallel osteotomies to form a 1.5 mm fracture gap or by drilling a 2‐mm diameter hole to create a trephine defect through the right mandibular ramus. Defects were filled with cartilage harvested from tibia fracture calli of eGFP or LacZ reporter mice 7 days postfracture as done previoulsy.^11^ Control defects were left empty. Mandibles treated with eGFP cartilage received unstable critical‐sized defects, were harvested for analysis 7, 14, and 28 days postengraftment, and were paraffin embedded (*N* = 3/time point). Mandibles treated with LacZ cartilage received either unstable or stable critical‐sized defects. LacZ animals receiving unstabilized defects were harvested at 14 and 28 days postengraftment (*N* = 3/time point). LacZ animals receiving stabilized defects were harvested at the same time points (*N* = 2/time point). Progression of healing was assessed via standard histology staining (HBQ) as above. HBQ histology from samples harvested 28 days postengraftment was assessed using Image J/Fiji software to determine the relative callus composition of bone, cartilage, and marrow. Randomly selected ×10 and ×20 images were analyzed. The region of interest was outlined and the image cropped to remove any extraneous tissue surrounding the fracture callus. The Fiji Color Threshold feature was used to estimate tissue volume in pixels. The Fiji thresholding method was set to default, the threshold color set to red, and the color space selected as HSB. For each image, the brightness was adjusted to include all possible pixels within the region of interest. Hue and saturation were adjusted to best approximate the HBQ staining patterns of bone, cartilage, and marrow for each sample. Pixel measurements for total callus, bone, cartilage, and marrow were recorded for each image. To confirm the accuracy of these measurements, bone, cartilage, and marrow pixels were summed and compared to the total callus pixels for each image. On average the sum was equivalent to 94% of the total callus pixel count. Raw pixel counts were used to calculate % total pixels (*N* = 2 images/animal). Donor versus host‐derived bone was determined via X‐gal staining as done previously.^11^ X‐gal staining was used to detect β‐galactosidase on frozen sections post‐fixed in 0.2% glutaraldehyde for 15 min, and then exposed to X‐gal staining solution overnight at 37°C.

### 2.7 Statistics

Statistical significance was determined using Prism software. For analyses involving more than two treatment groups, one‐way analysis of variance followed by Tukey's nonparametric multiple comparisons test was performed. For analyses involving only two treatment groups, either the Mann‐Whitney nonparametric or the unpaired, parametric *t* test was performed. *p* ≤ .05 was designated as statistically significant.

## 3 RESULTS

### 3.1 Mandible fracture models produce distinct reparative patterns

Unstable fractures were generated by creating a complete osteotomy through the right mandibular ramus from the anterior border of the coronoid process to the anterior border of the angular process (Figure 1A). Stable fractures were created by drilling a 1‐mm diameter trephine defect through the right mandibular ramus in an anatomically similar location as the unstable osteotomy (Figure 1B). In comparing these two fracture models, histological analysis revealed that unstable fractures developed a robust cartilage callus by 10 days postfracture (Figure 1E,F); whereas, stable fractures exhibited little to no cartilage at all time points (Figure 1I‐N). Cartilage was only observed in the stable fracture models at 10 days postfracture. This was the time point at which the greatest amount of cartilage was observed in both fracture models. Total callus size, absolute bone and absolute cartilage volume were significantly lower in stable fractures than in unstable fractures. This was quantitatively confirmed via stereological analysis of fracture calli harvested at 10 days postfracture (Figure 1O). Three out of the five stable fractures collected at day 10 displayed absolutely no cartilage at this time point. For the samples that did, small amounts of cartilage were found just outside the defect site, usually ventral and posterior to the defect (Figure 1K, yellow arrowhead). Cartilage was never observed within the defect in the stable model, whereas the majority of the cartilage callus was found between the fractured bone ends of unstable fractures. We believe the small regions of cartilage occasionally seen in the stable model are most‐likely due to motion derived from residual bone chips left behind from drilling the stable fracture. Indeed, although the fracture site was flushed multiple times with saline following trephine drilling, bone chip remnants were observed adjacent to the fracture site and were often surrounded by small regions of cartilage staining positive for Alcian blue. Unstabilized fractures reached complete bony‐bridging by 21 days postfracture (Figure 1G,H), and stabilized fractures were completely bridged by 10 days postfracture (Figure 1K,L).

### 3.2 Mandibular chondrocytes transform into osteoblasts during endochondral repair

The unstabilized mandible fracture model was used to assess the role of chondrocyte‐to‐osteoblast transformation during mandibular endochondral repair. Samples were analyzed at 14 days postfracture, the time point during which we have found the most significant cartilage‐to‐bone replacement occurs in tibia.^8^ Chondrocyte lineage tracing was performed using a tdTomato reporter expressed under the control of a cartilage‐specific, inducible Aggrecan‐Cre^ERT2^ promoter (Figure 2 and S2). The vast majority of chondrocytes within the fracture callus were positive for tdTomato, indicating that Cre‐recombination was highly efficient (Figure 2C). Robust tdTomato expression was also observed in osteoblasts and bone‐lining cells within the newly formed bone, demonstrating that these cells were chondrocyte‐derived (Figure 2F). Low magnification images of the transition zone region, where callus cartilage joins the newly formed bone, indicate that the new bone is highly vascularized and that chondrocytes contribute significantly to bone formation (Figure 2A‐E).

### 3.3 Mandibular callus chondrocytes in the transition zone have a transient phenotype

**Figure 3:**
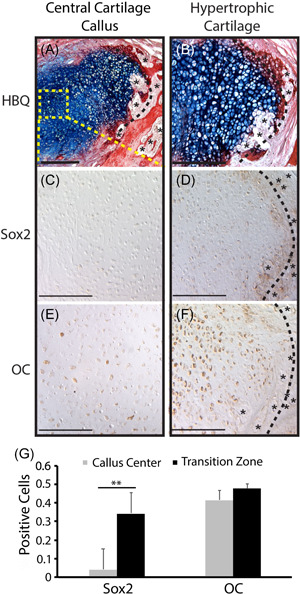
Hypertrophic chondrocytes at the mandibular fracture callus transition zone express stem cell and osteogenic markers. C57BL/6 mandibles were given unstable osteotomies and harvested 10 days postfracture. HBQ (A, B) histology shows a robust TZ (black dotted line) region within the fracture callus, marked by newly formed blood vessels (*) of the invading vasculature at the chondro‐osseous junction. Immunohistochemical analysis demonstrates that hypertrophic chondrocytes at the TZ express the stem cell marker *Sox 2*(D) as well as the classic osteogenic marker *Osteocalcin*(F). Chondrocytes at the center of the cartilage callus and distant from the TZ (boxed region in A) either do not express these genes or express at a significantly lower level (C, E). Quantification of % positively staining cells (G) reveals a statistically significantly difference in staining patterns between these two regions for Sox2. However, a statistically significant difference was not noted for OC. This is likely due to the inclusion of cells with even faint staining as positive. Qualitative comparison of the two regions demonstrates a consistent difference in the strength of staining. HBQ: cartilage is blue, bone is red. *N* = 5/antibody. Scale = 200 µm. HBQ, hall brunt quadruple; OC, osteocalcin; TZ, transition zone [Color figure can be viewed at wileyonlinelibrary.com]

The greatest amount of callus cartilage was observed in unstable mandible fracture samples harvested 10 days postfracture with a robust transition zone observed in all samples (Figure 1E and 3A,B). Phenotype of the chondrocytes within the fracture callus were evaluated in two distinct regions: (1) in the central callus, away from the newly forming bone and invading vasculature, or (2) the hypertrophic chondrocytes at the transition zone. Sox2 immunohistochemistry revealed minimal expression of this transcription factor that is associated with stemness in the central cartilage callus (4% of chondrocytes). In contrast, statistically significant staining was observed in hypertrophic chondrocytes at the transition zone region (35% of chondrocytes, Figure 3C,D,G). The strongest Sox2 expression was in cells adjacent to blood vessels or marrow space (Figure 3D*). The bone marker, OC, was expressed most strongly in the hypertrophic chondrocytes at the Transition Zone with more lower, but distinct staining found in the central cartilage callus (Figure 3E‐G). A statistically significant difference in regional OC was not detected upon cell count, likely due to the protocol used to count positively staining cells irrespective of staining intensity.

### 3.4 Cartilage grafts promote vascularized bone regeneration in critical‐sized mandibular defects

**Figure 4:**
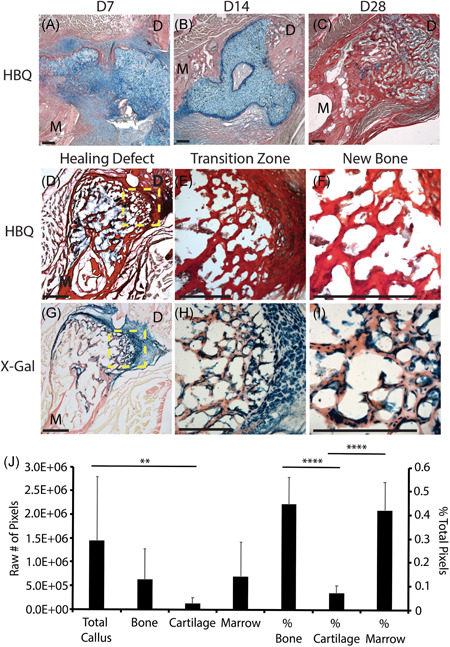
Cartilage engraftment results in complete bony‐bridging of critical‐sized mandibular defects through graft‐derived bone formation. HBQ shows gradual integration of cartilage grafts with the surrounding bone of critical‐sized unstable defects at 7 (A) and 14 (B) days postengraftment. Complete bony‐bridging of defects occurred by 28 days postengraftment (C). Newly formed bone shows a robust marrow cavity, indicating healthy vascularization of the new bone (C). Quantification of callus composition at 28 days postengraftment demonstrates that nearly the entire cartilage graft converted to bone by this time point (J). Only 7% of the total callus composition consists of cartilage at 28 days postengraftment. This is statistically significantly less than that of bone or marrow (J). X‐gal staining at D28 postengraftment (G–I, corresponds to HBQ in D–F) clearly identifies host versus donor‐derived tissues (G) and reveals that osteoblasts and bone‐lining cells of the newly formed bone are graft‐derived (I). Low magnification images of the defect site (D, G) demonstrate the significant contribution of engrafted cartilage to newly formed bone, especially at the transition zone (E, H, boxed regions in D, G). HBQ: cartilage is blue, bone is red. X‐gal: donor cells are blue. *N* = 3–6/fracture type/time point. Scale = 200 µm. **p < .01. ****p < .0001. D, distal; HBQ, hall brunt quadruple; M, mesial [Color figure can be viewed at wileyonlinelibrary.com]

Due to the important role of chondrocytes during mandibular endochondral repair, we tested the ability of cartilage grafts to heal critical‐sized mandibular defects using two different models: (1) an unstable 1.5 mm defect created by making two parallel osteotomies and (2) a stable 2‐mm diameter trephine defect. Defects were confirmed to be critical‐sized since no bony bridging was observed in empty defect controls for either model (Figure S1). HBQ histology revealed that engrafted cartilage began to integrate with the surrounding bone at 7 days postengraftment, cartilage integration was robust by 14 days postengraftment, and complete bony‐bridging of the defect was observed by 28 days postengraftment in both models (Figure 4A‐C). Newly formed bone had a robust marrow cavity, indicating healthy and highly vascularized new bone (Figure 4C). Quantification of HBQ histology using Image J/Fiji Color Threshold software revealed that the vast majority of the graft converted from cartilage to trabeculated bone by 28 days postengraftment with an average of only 7% of the cartilage remaining within the defect at that time point. Conversely, quantification confirmed that robust trabecular bone formation was observed within the grafted region with statistically more bone tissue and marrow space compared to cartilage (>40% each, *p* < .0001, Figure 4J). Chondrocytes from the engrafted cartilage as well as osteoblasts and bone‐lining cells in the newly formed bone were X‐gal positive, indicating their donor origin (Figure 4G‐I). Donor‐derived bone (X‐gal positive) was clearly distinguished from host‐derived bone (X‐gal negative) (Figure 4G). Due to the delicacy of the cryoembedded tissues, quantification of donor versus host cells was not possible. However, as nearly the entire span of newly formed bone within the defect site was X‐gal positive and X‐gal staining was limited to within the donor region, this suggests not only that cartilage graft chondrocytes contributed the majority of newly formed bone but also that the X‐gal staining was highly specific.

## 4 DISCUSSION

Despite differences in embryonic origin and developmental mechanism, our study demonstrates that mandibular fractures proceed through conserved mechanisms of repair as seen in long bones. One of the basic tenants of long‐bone fracture repair is that mechanical motion regulates the mechanism of healing, such that motion promotes cartilage formation and endochondral ossification whereas rigid stabilization results in direct bone formation through intramembranous ossification.^21^ In this study we confirm that motion within the mandible plays a similar, dominant role in directing the mechanism of bone formation during fracture repair (Figure 1). Although small regions of cartilage were sometimes observed in our stable fracture samples, cartilage formation always occurred outside of the trephine defect and was associated with the presence bone chips. This correlates to tibia fracture studies that show an absence of cartilage with absolute ridged fixation resulting in a model of intramembranous repair.^21^

One observation from our study was that stabilized fractures healed faster than those that were unstabilized. Whereas complete bony‐bridging was observed in stable fractures at 10 days postfracture, unstable fractures completely bridged at 21 days postfracture. The fracture models used in this study differed in both size and shape (Figure 1). Thus, the difference in healing time is likely an artifact of the different fracture designs rather than the result of an intrinsic difference in the rate of healing between endochondral and intramembranous ossification. Indeed, Thompson et al.^21^ demonstrated using unstabilized versus stabilized transverse tibia fracture models that the time required to achieve bony bridging is identical when bones heal through endochondral versus intramembranous ossification. One limitation of our study is that we did not evaluate the quality of regenerated bone. The degree of bone repair was solely determined by the degree of bone formation and bony bridging through histology.

There has been a centuries‐long debate regarding the fate of callus chondrocytes during endochondral repair. In the early 1800s cartilage was believed to transform into bone.^22^, ^23^, ^24^ However, in the mid‐1800s Muller and Sharpy reported that all chondrocytes ultimately undergo apoptosis and that bone is derived from a separate population of osteoprogenitors.^22^ The development of modern murine genetics has enabled accurate lineage tracing and the determination of cell fate. Recent genetic evidence now demonstrates that although some callus chondrocytes undergo apoptosis to make room for the marrow cavity, a significant portion of chondrocytes transform into osteoblasts and directly contribute to the formation of new bone.^8^, ^9^, ^10^, ^12^, ^13^ This has been confirmed using a variety of Cre lines and genetically labelled transplants in the contexts of long‐bone development, growth, and repair as well as mandibular condylar development.^8^, ^9^, ^10^, ^11^, ^12^, ^13^, ^14^ Here we demonstrated via Aggrecan‐Cre^ERT2^ lineage tracing that mandibular callus chondrocytes undergo a similar chondrocyte‐to‐osteoblast transformation during mandibular fracture repair and that callus chondrocytes significantly contribute to the formation of new bone (Figure 2).

Limited work has been done to investigate the mechanisms that regulate chondrocyte‐to‐osteoblast transformation. Previous work in long bone fracture repair demonstrates that hypertrophic chondrocytes undergoing chondrocyte‐to‐osteoblast transformation at the Transition Zone express genes traditionally associated with stem cell pluripotency (*Sox2*, *Oct4*, *Nanog*) as well as classic osteogenic genes (*Runx2*, *Osx*, *OP*, *OC*, *Col1*), suggesting that chondrocytes may undergo stem cell‐like plasticity before differentiating into osteoblasts.^8^, ^11^ *Sox2* and *β‐catenin* have been shown to play critical roles in regulating chondrocyte transformation. Hu et al.^8^ demonstrated that deletion of *Sox2* in *Sox2* ^*CreERT2/fl*^ mice during tibia fracture repair significantly reduced new bone formation. Houben et al.^13^ reported similar findings following deletion of β‐catenin using the Col10a1‐Cre driver during endochondral bone development. Furthermore, Wong et al.^7^ demonstrated using the Aggrecan‐Cre^ERT2^ driver that β‐catenin is critical to chondrocyte‐to‐osteoblast transformation and hence cartilage‐to‐bone conversion during endochondral fracture repair.

In this study we looked to understand if phenotypic modulation of mandibular chondrocytes paralleled that seen in long bone fracture repair. Evidence presented here suggests further similarity in the regulatory mechanisms of craniofacial and appendicular bones during fracture repair. The transition zone histomorphometry of mandibular fractures was similar to that seen in previously published tibia fracture studies (Figure 3A,B).^8^, ^9^, ^10^, ^11^, ^12^ Furthermore, mandibular chondrocyte gene expression in the hypertrophic region at the transition zone paralleled that seen previously in tibial fracture studies, with chondrocytes expressing the stem cell marker Sox2 and the classic osteogenic marker OC (Figure 3). It is important to note that Sox2 expression is limited almost exclusively to hypertrophic chondrocytes at the transition zone and that expression is absent or significantly lower in pre‐hypertrophic chondrocytes (Figure 3). Similarly, OC expression is significantly stronger at the transition zone compared to the central callus. Although our cell count did not reflect a statistically significant difference in expression between these two regions for OC, this is likely due to our inclusion of cells with any degree of staining in our positive count. Qualitative comparison of IHC in these two regions consistently shows lighter staining in the central callus and robust staining at the transition zone. Future studies to understand the temporal expression or potential coexpression of Sox2 and OC would advance our understanding of the molecular sequence in both the mandible and tibia.

Autologous bone grafts are the current gold standard of care. In the case of mandibular reconstruction, nonvascularized grafts are traditionally harvested from the rib or iliac crest, whereas vascularized free flaps are primarily harvested from the fibula.^25^ Due to the necessity of the vasculature for graft survival, nonvascularized bone grafts are only used to treat small defects where a robust vascularized tissue bed is already present. A flap is required in cases of soft tissue injury.^25^ Although autologous bone grafts have had significant clinical success, they require a second surgical site and are accompanied by notable costs including donor site morbidity and limited supply.^11^, ^18^ Bone allografts have been developed as an alternative graft material and are readily available; however, they have significantly reduced bioactivity and a high rate of clinical failure due to poor osteointegration and osteonecrosis of the graft.^19^ As an avascular tissue, cartilage is not only capable of surviving without a vascular supply but is also responsible for recruiting blood vessel invasion for the newly formed bone during endochondral ossification.^2^, ^3^ Thus, cartilage is not only better suited than bone to survive within poorly vascularized areas of tissue injury but its use also parallels the natural healing process of endochondral repair.

There has been increasing interest in the development of cartilage‐based therapies.^11^, ^26^, ^27^, ^28^, ^29^ Bahney et al.^11^ demonstrated that cartilage grafts heal critical‐sized tibial defects and that a significant portion of newly formed bone is donor‐derived. Furthermore, they demonstrated that human bone marrow‐derived mesenchymal stem cells (hMSCs) can be differentiated and cultured into viable cartilage grafts for transplant.^11^ Other studies have also demonstrated the efficacy of using cartilage to engineer bone.^27^, ^28^, ^29^ Scotti et al.^29^ demonstrated that subcutaneous implantation of hypertrophic cartilage derived from hMSCs gives rise to an ectopic “bone organ” with a structure and functionality comparable to that of native bones. Dang et al.^28^ reported that critical‐sized calvarial defects can heal through endochondral ossification when treated with hMSCs pushed toward chondrogenesis through the controlled release of transforming growth factor β1 and bone morphogenetic protein‐2 delivered via bioactive microparticles. And Cunniffee et al.^27^ showed that decellularized hypertrophic cartilage freeze‐dried to generate porous scaffolds can produce complete bony bridging of critical‐sized femoral defects. Our data support that cartilage is an effective tissue graft to promote bone regeneration in the mandible in both stabilized and unstabilized fracture contexts (Figure 4). Although bony bridging was achieved using both models, stabilized critical‐sized defects were much better at retaining the cartilage graft within the defect than unstabilized defects. With the unstabilized model, the cartilage graft tended to pop out of the defect and remain within the surrounding musculature. Thus, cartilage grafts were more effective in producing bony bridging in the stabilized model due to increased graft retention. Quantification of callus composition at 28 days postengraftment revealed that nearly the entire engrafted cartilage converted to bone by this time point with only 7% callus composition consisting of cartilage. One limitation of this study was our inability to quantify donor versus host cells due to the delicacy of the cryoembedded tissues. This is a potential area for future development. Despite this limitation, X‐gal staining was highly specific as demonstrated by the presence of X‐gal staining only within the donor region. The amount of bone that was X‐gal positive continuously spanned nearly the entire length of the defect in all of our samples, suggesting that cartilage graft chondrocytes contributed the majority of newly formed bone.

Identification of host versus donor tissues demonstrates that healing occurs through the transformation of engrafted chondrocytes to osteoblasts that form the new bone. It is unlikely that the contribution of donor cells to newly formed bone was due to contamination of cartilage grafts by a separate stem cell population. Our lab has performed extensive analysis on the composition of tissues harvested using this methodology.^11^ Previous analysis of grafts reveals nearly pure cartilage. In situ hybridization and qPCR analysis reveals significantly higher expression of cartilage markers (Sox9, Col2, Col10) and nearly nonexistent expression of the osteoblast marker OC compared to total callus.^11^ Furthermore, the role of engrafted chondrocytes in contributing to new bone formation is corroborated by our lineage tracing analysis, which demonstrates the contribution of Aggrecan‐expressing chondrocytes to osteoblast and bone‐lining populations.

Our data demonstrate that the ability of cartilage to heal a critical‐sized defect is not limited by the endogenous healing pathway and is effective for vascular bone regeneration. Importantly, since complete mandibular healing was attained through the use of tibia‐derived cartilage, our data indicates that the embryonic origin or mode of tissue development does not significantly impact the efficacy of graft tissues (Figure 4).

## 5 CONCLUSION

Although craniofacial bones and long bones have significant developmental differences, our study supports that these bones heal via conserved mechanisms that reflect recent advances in our understanding of long bone fracture repair. Notably, during endochondral fracture repair, both sets of bones rely on the significant contribution of chondrocytes for the formation of new bone through chondrocyte‐to‐osteoblast transformation. This data provides important insight into the enhancement of craniofacial fracture therapies, such as the use of cartilage as a graft tissue, and it supports the application of findings from appendicular fracture studies to the treatment of craniofacial fractures.

## CONFLICT OF INTERESTS

Dr. Theodore Miclau III discloses Board or committee positions for the AO Foundation, Foundation of Orthopaedic Trauma, Inman Abbott Society, International Combined Orthopaedic Research Societies, International Orthopaedic Trauma Association, Orthopaedic Research Society, Orthopaedic Trauma Association, Osteosynthesis and Trauma Care Foundation, and San Francisco General Hospital Foundation. He has received research support from Baxter and is a Paid consultant for Arquos, Bone Therapeutics, NXTSENS, Surrozen, and Synthes with stock or stock options at Arquos. None of the paid positions are related to the work presented in this manuscript. Dr. Ralph S. Marcucio has an unpaid position on the Board of Directors for American Association of Anatomists and the International Section of Fracture Repair (ISFR) through the Orthopaedic Research Society (ORS). He is also on the editorial board for the Journal of Orthopaedic Research and Developmental Biology. These disclosures do not represent a competing interest with the publication of this manuscript. Dr. Chelsea S. Bahney discloses an unpaid Board or committee positions on the Board of Directors for Orthopaedic Research Society (ORS), Tissue Engineering and Regenerative Medicine International Society (TERMIS), the International Section of Fracture Repair (ISFR), and the Orthopaedic Trauma Association (OTA). Further, Dr. Bahney is a paid employee of the nonprofit Steadman Philippon Research Institute (SPRI). SPRI exercises special care to identify any financial interests or relationships related to research conducted here. During the past calendar year, SPRI has received grant funding or in‐kind donations from Arthrex, DJO, MLB, Ossur, Siemens, Smith & Nephew, XTRE, and philanthropy. These funding sources provided no support for the work presented in this manuscript. No other authors have any conflicts of interest to report.

## AUTHOR CONTRIBUTIONS

Sarah A. Wong, Theodore Miclau III, Ralph S. Marcucio, and Chelsea S. Bahney contributed to study conception. Sarah A. Wong, Ralph S. Marcucio, and Chelsea S. Bahney designed the experiments. Sarah A. Wong, Diane P. Hu, Charles Lam, Joshua Slocum, and Michael Nguyen executed the experiments. Sarah A. Wong, Diane P. Hu, Ralph S. Marcucio, and Chelsea S. Bahney conducted data analysis and interpretation. Sarah A. Wong, Theodore Miclau III, Ralph S. Marcucio, and Chelsea S. Bahney provided financial support and materials. Sarah A. Wong prepared initial draft of manuscript. All authors have read and approved the final submission.

## Supporting information

Supporting information.

Supporting information.

Supporting information.
