# Supplementary figures and images for: Chondrocyte‐to‐osteoblast transformation in mandibular fracture repair
Source: J Orthop Res. 2020 Nov 18;39(8):1622–32. doi: 10.1002/jor.24904 (PMC8451921; doi:10.1002/jor.24904)

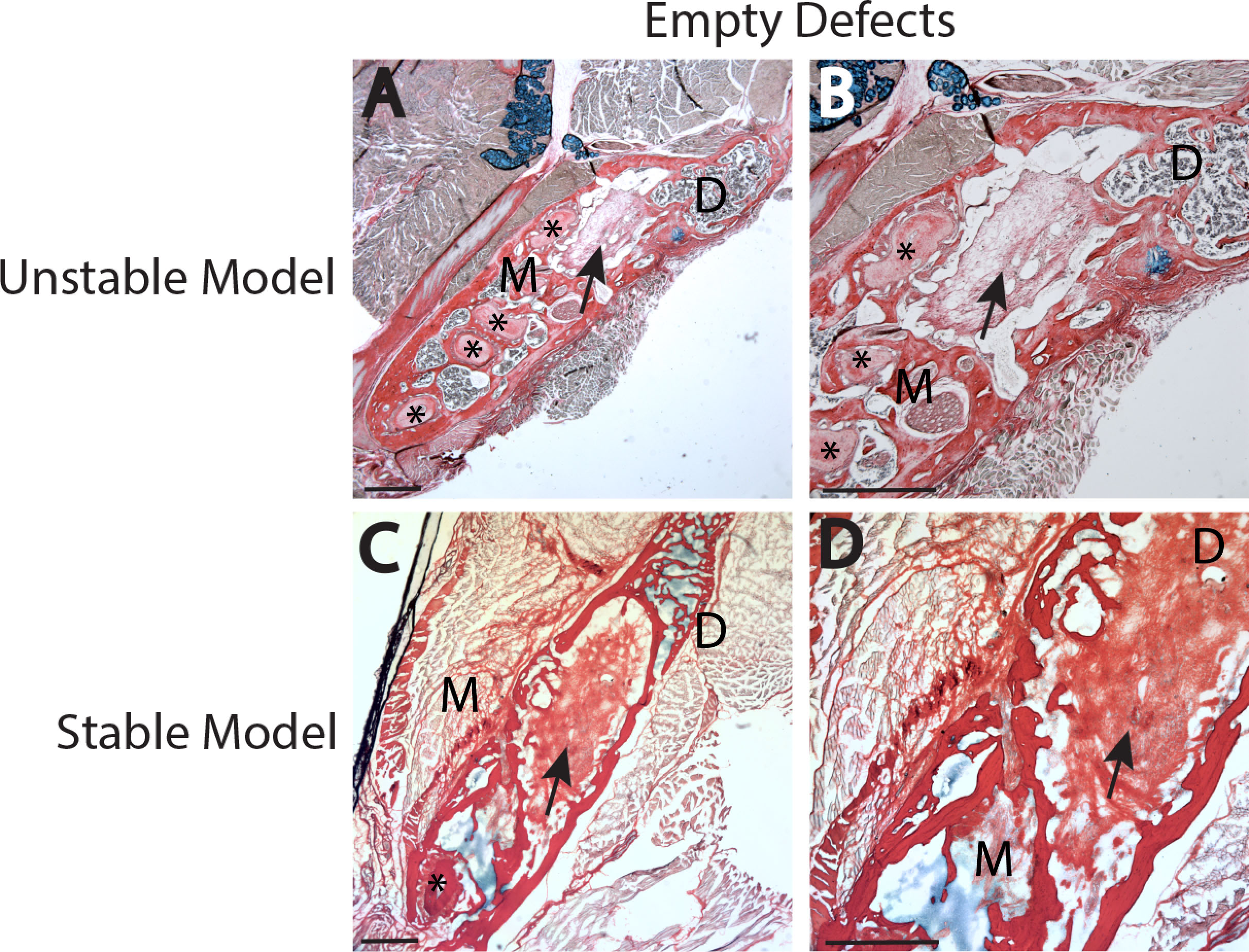

Supplement: Supplementary file 1 — Supporting information. [file JOR-39-1622-s002.jpg]

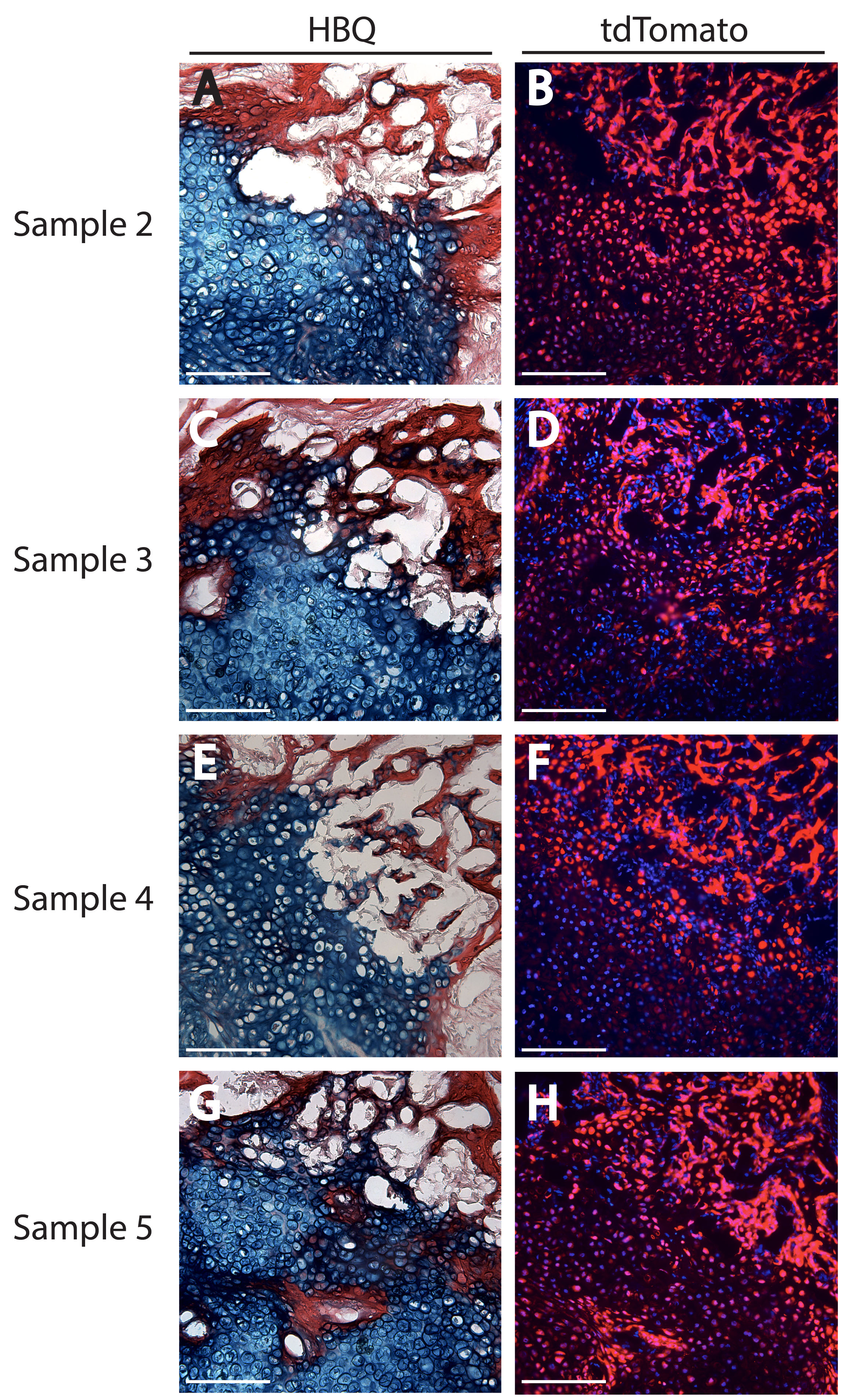

Supplement: Supplementary file 2 — Supporting information. [file JOR-39-1622-s003.jpg]
