# Supplementary material for: Chondrocyte‐to‐osteoblast transformation in mandibular fracture repair
Source: J Orthop Res. 2020 Nov 18;39(8):1622–32. doi: 10.1002/jor.24904 (PMC8451921; doi:10.1002/jor.24904)
Supplement: Supplementary file 3 — Supporting information. [file JOR-39-1622-s001.docx]

**Supplemental Figure 1**: **Confirmation that Defects are Critical-Sized**

Hall Brunt Quadruple/HBQ histology reveals that empty defects, both unstable **(A-B)** and stable **(C-D)**, are critical-sized. Defects remain unbridged and fill with fibrous tissue **(arrows)** at 28 days post-fracture, the latest harvest time point. HBQ: cartilage is blue, bone is red. M = Mesial. D = Distal. (*) = Mandibular molar root. Scale=500 µm.

**Supplemental Figure 2**: **Lineage Tracing Data for Remaining Samples**

HBQ histology **(A, C, E, G)** and lineage tracing analysis using the Aggrecan-Cre^ERT2^ driver and Ai9 tdTomato reporter mouse **(B, D, F, H)** demonstrate robust tdTomato expression in callus chondrocytes as well as within osteoblasts of the newly formed bone at the Transition Zone. This pattern of tdTomato expression is consistent across samples. DAPI counterstain was used to visualize nuclei (blue). N=5. Scale=100 µm.
